# Exocyst complex regulates fungal-mediated IL-33 release from cancer cells

**DOI:** 10.1038/s41392-026-02629-0

**Published:** 2026-03-06

**Authors:** Aftab Alam, Shyamananda Singh Mayengbam, Scott I Abrams, Jun Qu, Prasenjit Dey

**Affiliations:** 1 Department of Immunology, Roswell Park Comprehensive Cancer Center, Buffalo, NY USA; 2 Department of Pharmaceutical Sciences, State University of New York at Buffalo, Buffalo, NY USA

**Keywords:** Innate immunity, Infection

**Dear Editor**,

Recent studies show that mycobiomes are pervasive in tumors and play a key role in reprogramming the tumor microenvironment.^1^ However, the mechanism of intratumor mycobiome-mediated tumorigenesis remains poorly understood and is an evolving area of research. Previously, we have shown that intratumor mycobiome facilitates IL-33 release from Kras-driven pancreatic ductal adenocarcinoma (PDAC) tumor cells, thereby attracting T helper 2 (T_H_2) cells and group 2 innate lymphoid cells (ILC2s) in the tumor microenvironment and promoting tumorigenesis.^2^ However, the mechanisms underlying fungal-mediated IL-33 release from PDAC cells remain unknown.

The alarmin, IL-33, a member of the IL-1 family of cytokines, is constitutively expressed and released by damaged or necrotic barrier tissues.^3^ IL-33 is a nuclear cytokine that requires an environmental stimulus to be secreted from cells. First, to determine the mechanism of IL-33 release from PDAC cells, we studied IL-33 compartmentalization in PDAC cells. IL-33 is a nuclear protein, and immunofluorescence (IF) analysis confirmed that IL-33 is localized in the nucleus of pancreatic cancer cells at the steady state (Fig. 1a). To stimulate IL-33 release, we treated PDAC cells with *A. alternata* extract. An IL-33-specific ELISA using spent media shows an increase at 2, 3, and 6 hours post *A. Alternata* treatment (Fig. 1a). Based on this data, we hypothesized that the IL-33 release mechanism utilizes a unique cell-secretion machinery and sought to identify it through protein-protein interaction studies. To delineate the IL-33 release pathway, we performed proteomic analyses to interrogate the IL-33 interactome in the PDAC cells. We conducted IL-33 immunoprecipitation (IP) experiments using nuclear and cytoplasmic extracts from PDAC cancer cells. Proteomics analysis revealed IL-33 interacting partners in the cytoplasm and in the nuclear extracts (Data not shown). Based on our interest in secretion pathways that enable IL-33 release, we focused on proteins known to be associated with protein transport. We examined the cytoplasmic and nuclear fractions for proteins that directly affect protein transport and identified three members of the exocyst family, EXOC2, EXOC4, and EXOC8,^4^ along with Rab11, a vital component of the exocytosis machinery,^4^ in the cytoplasmic fraction of the IL-33 pull-down (Fig. 1a).

**Fig. 1:**
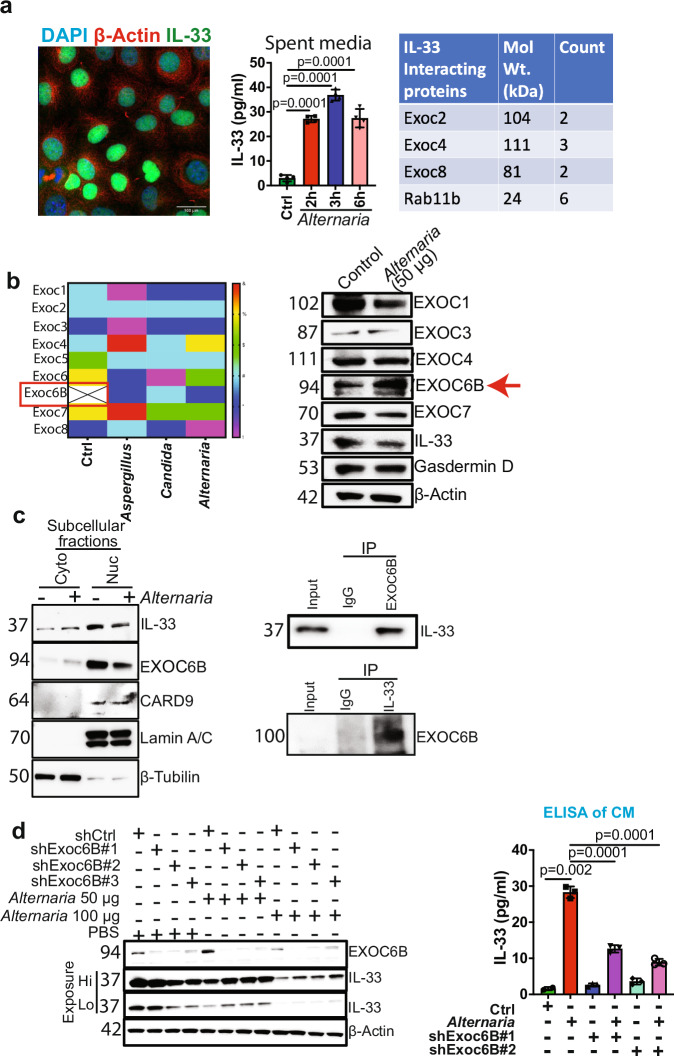
**a** Fluorescence image showing nuclear expression of IL-33 (green) in AK-B6 PDAC cell line. β-actin (red) was used as a cytoplasmic stain, and DAPI (blue) was used for nuclear staining; magnification, 40×. IL-33 was measured in the spent medium using ELISA in AK-B6 PDAC cell line treated with *A. alternata* extract for different time points (2, 3, and 6 h) (*n* = 3). Table showing Exocyst complex proteins EXOC2, EXOC4, EXOC8, and Rab11b were identified by proteomics analysis as IL-33 interacting partners. **b** Heat map showing exocyst complex protein expression in AK-B6 cells treated with control, *Aspergillus fumigatus, Candida albicans*, and *A. alternata*. Western blot analysis of AK-B6 cells treated with *A. alternata* to check the expression of EXOC1, EXOC3, EXOC4, EXOC6B, EXOC7, IL33, Gasdermin-D, and β-actin was used as the loading control. **c** Representative western blots demonstrating the subcellular fractionation of AK-B6 PDAC cell line treated with or without *A. alternata* extract showing IL-33 and EXOC6B expression in cytoplasm and nucleus. Lamin A/C and β-tubulin were used as nuclear and cytoplasmic loading controls, respectively. Co-Immunoprecipitation of EXOC6B and IL-33 in AK-B6 PDAC cells to study their interaction. **d** Western blot analysis of *A. alternata* extract-treated AK-192 control and AK-192 EXOC6B knockdown PDAC cells. IL-33 was measured in the spent medium using ELISA in the AK-192 and AK-192 EXOC6B knockdown PDAC cell lines treated with *A. alternata* extract for 3 h (*n* = 3)

The exocyst complex consists of eight subunits, EXOC1-EXOC8, which function as a tethering complex to carry the secretory cargo to the plasma membrane and facilitate exocytosis.^4^ Individual components of the exocyst complex, such as EXOC7, are shown to drive a pro-tumorigenic secretome and promote cancer progression.^5^ The above data demonstrates that IL-33 interacts with the exocytosis complex proteins (EXOC2, EXOC4, and EXOC8) at a steady state.

To understand the fungal-mediated release of IL-33, we performed a second proteomic analysis of whole-cell lysates of PDAC cancer cells upon treatment with the *A. alternata, Candida albicans (C. albicans)*, and *Aspergillus fumigatus (A. fumigatus)* extracts (Fig. 1b). We identified eight exocyst complex proteins in the fungal-treated samples, and EXOC6B was highly upregulated in the fungal-treated groups (Fig. 1b). We validated the expression of the exocyst protein complex upon *A. alternata* extract treatment, which is known to trigger IL-33 release from PDAC cells.^2^ Our results revealed that EXOC6B expression increased after *A. alternata* treatment (Fig. 1b). Whereas the expression of EXOC1 and EXOC7 decreased, EXOC3, and EXOC4 remained unchanged (Fig. 1b). Importantly, Gasdermin D (GSDMD), a known mediator of IL-33 release, remains unaltered upon *A. alternata* extract treatment, indicating the involvement of a non-canonical pathway for fungal-mediated IL-33 release (Fig. 1b).

We further examined the compartmentalization of EXOC6B in PDAC cells through cell fractionation to better understand the nucleocytoplasmic transport of IL-33. Western blot analysis showed that EXOC6B, CARD9 and IL-33 were more abundant in the nuclear extract than in the cytoplasmic extract, and treatment with *A. alternata* reduced the nuclear localization of both IL-33 and EXOC6B (Fig. 1c), indicating that the fungal extract enhances the nucleocytoplasmic transport of both proteins in PDAC cells. To further understand the involvement of EXOC6B in the IL-33 release pathway, we examined the interaction of EXOC6B with IL-33 in PDAC cancer cells. We performed IP in whole-cell lysates from PDAC cancer cells using an anti-IL-33 antibody, followed by Western blot with an anti-EXOC6B antibody, demonstrating IL-33 interaction with EXOC6B (Fig. 1c). Similarly, a reverse IP with an anti-EXOC6B antibody pulled down IL-33, confirming the interaction between IL-33 and EXOC6B (Fig. 1c), suggesting that the exocyst complex interacts with IL-33 and can drive its release from PDAC cells.

To further establish the mechanistic link between EXOC6B and fungal-mediated IL-33 release, EXOC6B was knocked down from the PDAC cancer cells, and treated with *A. alternata* at two different doses of 50 µg and 100 µg for 3 hours. Western blot analysis shows that EXOC6B knockdown leads to the accumulation of IL-33 inside the cells in the presence of *A. alternata* **(1** **d)**. Simultaneously, we observed decreased IL-33 release in the spent media, at a 50 µg dosage of *A. alternata* (Fig. 1d). Moreover, EXOC6B depletion and *A. alternata* treatment at a higher dose of 100 µg disrupted IL-33 release, albeit less effectively than at lower doses, indicating that EXOC6B is a major pathway for IL-33 release, although an alternate mechanism of release might be triggered at higher *A. alternata* concentrations. (Fig. 1d). These data indicate that EXOC6B facilitates the fungal-mediated release of IL-33 from PDAC cells.

In summary, this study revealed a previously undescribed molecular mechanism by which fungi mediate the release of IL-33 from PDAC cells. Specifically, we discovered that IL-33 release involves the exocyst complex, a vital membrane trafficking complex that regulates the cell secretome.^5^ The findings here significantly advance our knowledge of the cellular and molecular mechanisms that govern this release. The fundamental discovery is that upon stimulation with fungi, EXOC6B regulates IL-33 translocation from the nucleus to the cytoplasm, a critical step preceding its secretion from cells. The knowledge about the mechanism of extracellular release of IL-33 is critical, as this pathway might provide insights into the mechanisms of release of other alarmins.^3^ Identification of the exocyst pathway as an IL-33 release mechanism opens a new paradigm for therapeutic targeting of PDAC cancer progression.

## Supplementary information

Supplemental 1

## Data Availability

All data supporting the findings of this study are available within the article and supplementary information or from the corresponding author upon reasonable request. Upon request, mouse cell lines (PJ-B6-4291, PJ-B6-4298, and PJ-B6-4271) generated in the lab will be available.
