# Supplementary material for: Exocyst complex regulates fungal-mediated IL-33 release from cancer cells
Source: Signal Transduct Target Ther. 2026 Mar 6;11:83. doi: 10.1038/s41392-026-02629-0 (PMC12963430; doi:10.1038/s41392-026-02629-0)
Supplement: Supplementary file 1 — Supplemental 1 [file 41392_2026_2629_MOESM1_ESM.docx]

**Supplementary Materials for**

**Exocyst complex regulates fungal-mediated IL-33 release from cancer cells**

^1^Aftab Alam,^1^Shyamananda Singh Mayengbam, ^1^Scott Abrams, ^2^Jun Qu, and ^1, 3*^Prasenjit Dey

Correspondence to: Prasenjit Dey, Elm & Carlton Sts. CGP/BLSC-L5307, Buffalo, New York 14263, Office: (716) 845-1300 x5269, Fax: (716) 845-1322, email: [prasenjit.dey@roswellpark.org](mailto:prasenjit.dey@roswellpark.org)

**This PDF file includes:**

**Materials and Methods:**

**Nuclear and cytoplasmic fractionation:** Nuclear and cytoplasmic fractionation was performed according to the Cell Fractionation Kit - Standard (ab109719) from Abcam, following the manufacture’s standard protocol.

**LC-MS Analysis:** Liquid chromatography-tandem mass spectrometry (LC-MS/MS) was conducted using a trapping nano-flow LC-Orbitrap Astral MS system, which comprised a Dionex Ultimate 3000 nano LC system, a Dionex Ultimate 3000 gradient micro-LC system with a WPS-3000 autosampler, and an Orbitrap Astral mass spectrometer (Thermo Fisher Scientific, Waltham, MA). Peptides derived from ~500ng protein was loaded onto a trapping column (100 µm ID × 5 cm, packed with 5µm C18, CoAnn Technologies, Richland, WA), to selectively remove matrix components, and then were delivered onto an analytical LC column (75 μm ID × 30 cm, packed with 1.7 μm C18, CoAnn Technologies, Richland, WA) for sensitive and reproducible separation. Mobile phases A and B for analytical peptide separation were 0.1% FA in 2% acetonitrile, and 0.1% FA in 88% acetonitrile, respectively. The peptides were eluted at a flow rate of 350nL/min using the following gradient: 4-35% B for 39 min; 35% to 97% B for 0.1 min; and isocratic at 97% B for 3 min.

Mass spectrometry was operated in data-independent acquisition (DIA) mode. MS1 spectra were acquired in the m/z range of 380 to 980 at a resolution of 180k. The normalized Automatic Gain Control (AGC) target for MS1 was set at 500%, with a maximum injection time of 5 ms. Precursor ions were isolated using a 4-Th wide window and fragmented by higher-energy collision-induced dissociation (HCD) at a normalized collision energy of 25%. MS2 spectra were acquired in the Astral (AST) mass analyzer in the m/z range of 350 to 1500. The normalized AGC target was set at 200%, the maximum injection time was set to 5 ms.

**Protein Identification and Quantification**

The MS raw files were analyzed by searching against the Uniprot-SwissProt mouse database (accessed January 2024, containing 17,179 protein entries) using DIA-NN (v1.8.1) in library-free mode. Trypsin was selected as the protease with one allowed missed cleavage. Cysteine carbamidomethylation was set as a static modification, while methionine oxidation and N-terminal acetylation were included as variable modifications. The precursor and protein FDR% were set to 1%. Mass accuracy was set to 5 ppm for MS1 and 9 ppm for MS2. The scan window radius was set to 10. All other settings were kept at their default values.

**Coimmunoprecipitation, immunoblotting, and antibodies**:The cell culture medium was removed, and the cells were washed twice in ice-cold phosphate-buffered saline (PBS). Cells are scraped and transferred to an Eppendorf tube, followed by centrifugation at 1500 rpm for 5 min at 4°C. The pelleted cells were incubated in radio immune precipitation assay (RIPA) buffer with proteinase and phosphatase inhibitors for 30 min. Lysates were then collected and centrifuged at 12000 rpm for 15 min at 4°C. Protein concentration was measured using the DC Protein Assay Kit (Bio-Rad, 5000111). SDS–PAGE and immunoblotting were performed as described previously in pre-cast bis-Tris 4–12% gradient gels (Invitrogen) (Dey, Baddour et al. 2017). The following antibodies were used: IL-33 (R&D systems, AF3626); CARD9 (CST,12283S), EXOC1 (Novus Biologicals), EXOC4 (Novus Biologicals, NBP1-89597), EXOC6B (Novus Biologicals, NBP1-56692), EXOC7 (Novus Biologicals, NB100-94901), Gasdermin D (Cell Signaling, 39754), CARD9 (Cell signaling, 77568S), Lamin A/C (Cell signaling, 2032S), β-Tubulin (Cell signaling, 2128S) and β-Actin (Sigma-Aldrich, A2228).IP experiments were done with 1 mg of protein from each whole-cell lysate were precleared with Protein A/G beads (Thermo Fisher Scientific) and then incubated with the indicated antibodies or control IgG overnight at 4 °C. After incubation, 50 µl of Protein G Agarose was added to each immune reaction and rotated overnight at 4 °C. The immune complex was washed three times with NP-40 buffer. Co-IP proteins were eluted by boiling for 5 min in 50 µl of Laemmli sample buffer containing 5% (vol/vol) 2-mercaptoethanol. Eluted proteins were resolved on a NuPage 4–12% BisTris gel and detected by IB using specific antibodies. Immunoreactive bands were detected by enhanced chemiluminescence (ECL Plus; Thermo Fisher Scientific) using horseradish peroxidase-linked anti-rabbit, anti-goat or anti-mouse secondary antibodies (Bio-Rad).

**shRNA knockdown:** shRNA knockdown was performed as described previously^2^. We screened 3-5 hairpins targeting the gene of interest and found two independent sequences that reduced mRNA levels by >60%. All the shRNA were purchased from Millipore Sigma. The shRNA sequences were as follows: EXOC6B: shRNA 5’-ATCCAGTGCTCCACGAATTTA-3’, 5’-AGGGCTTTGTGGACTCTATTA-3’, 5’-GAGGGCAAGGAACTGGTAATA-3’, 5’-GTACTCCAGCCTCCGTCTAAT-3

**Enzyme-linked immunosorbent assay (ELISA):** ELISA was done using cell culture spent media from PDAC mouse cell lines, tumor intestinal fluid (TIF) from mouse PDAC tumors and lung BAL fluid. Cell culture media was concentrated using Amicon Ultra centrifugal filter units (Millipore, Z717185) before conducting ELISA. IL-33 ELISA was performed using LEGEND MAX Mouse IL-33 ELISA Kit (Biolegend) using manufacturer’s standard protocol.

**Cell culture and establishment of primary PDAC lines:** The following cell lines are a gift from Dr. Ronald DePinho (AK-B6, AK192). All cell lines were routinely cultured in RPMI 1640 (Invitrogen) in 10% FBS (Invitrogen), 100 U/mL penicillin and 100 U/mL streptomycin. For inducible KPC derived cell lines, 1 mg/mL of doxycycline was directly added to the media. The cell lines were mycoplasma-free, based on tests done monthly in the laboratory using Lonza’s MycoAlert Mycoplasma Detection Kit assays, with confirmatory tests by PCR-based assays. *Alternaria alternata* extract (XPM1D3A2.5, Stallergenes Greer) treatment was done for 2, 3, and 6h at a dose of 50 or 100 μg.

For **immunofluorescence,** cells were stained with primary antibodies (IL-33, EXOC6B, and β-actin) overnight, followed by incubation with fluorescently labeled secondary antibodies at room temperature for 2 hrs. The nuclei are stained with DAPI. Immunofluorescence slides were imaged with a Leica confocal Microscope.

**Statistical Analysis**

GraphPad Prism software was used to analyze all data. Data are presented as mean ± SD. All quantitative results were assessed by an unpaired Student’s T-test after confirming that the data met appropriate assumptions (normality and independent sampling). The student *t*-test assumed two-tailed distributions to calculate statistical significance between groups. Unless otherwise indicated, for all *in vitro* experiments, three technical replicates were analyzed.
